# Supplementary material for: Reconstructing B cell lineage trees with minimum spanning tree and genotype abundances
Source: BMC Bioinformatics. 2023 Feb 27;24:70. doi: 10.1186/s12859-022-05112-z (PMC9972711; doi:10.1186/s12859-022-05112-z)
Supplement: Supplementary file 1 — Additional file 1: Fig. S1. ClonalTree configuration performance; Fig. S2. Performance comparison among GCtree, ClonalTree, and GLaMST using GED distances on three categories of trees; Fig. S3. Editing the reconstructed B cell lineage tree by adding unobserved internal nodes; Fig. S4. Editing the reconstructed BCR lineage tree to reduce the depth of the tree while keeping its overall cost; Fig. S5. An example of MRCA calculation; Fig. S6. An example of COAR calculation for a given leaf. [file 12859_2022_5112_MOESM1_ESM.pdf]

Reconstructing B cell lineage trees with minimum spanning tree and genotype abundances
Supplementary Figures

Nika Abdollahi[1,3], Lucile Jeusset[1,2], Anne de Septenville[2], Frédéric Davi[2] and Juliana Silva Bernardes[1,*]

[1]Sorbonne Université, CNRS, UMR 7238, Laboratoire de Biologie Computationnelle et Quantitative, Paris, France

[2]Sorbonne Université, AP-HP, Hôpital Pitié-Salpêtrière, Department of Biological Hematology, Paris, France

[3]IMGT®, the international ImMunoGeneTics Information System, CNRS, Institute of Human Genetics, Montpellier, France

[*]To whom correspondence should be addressed; E-mail: juliana.silva_bernardes@upmc.fr.

November 30, 2022

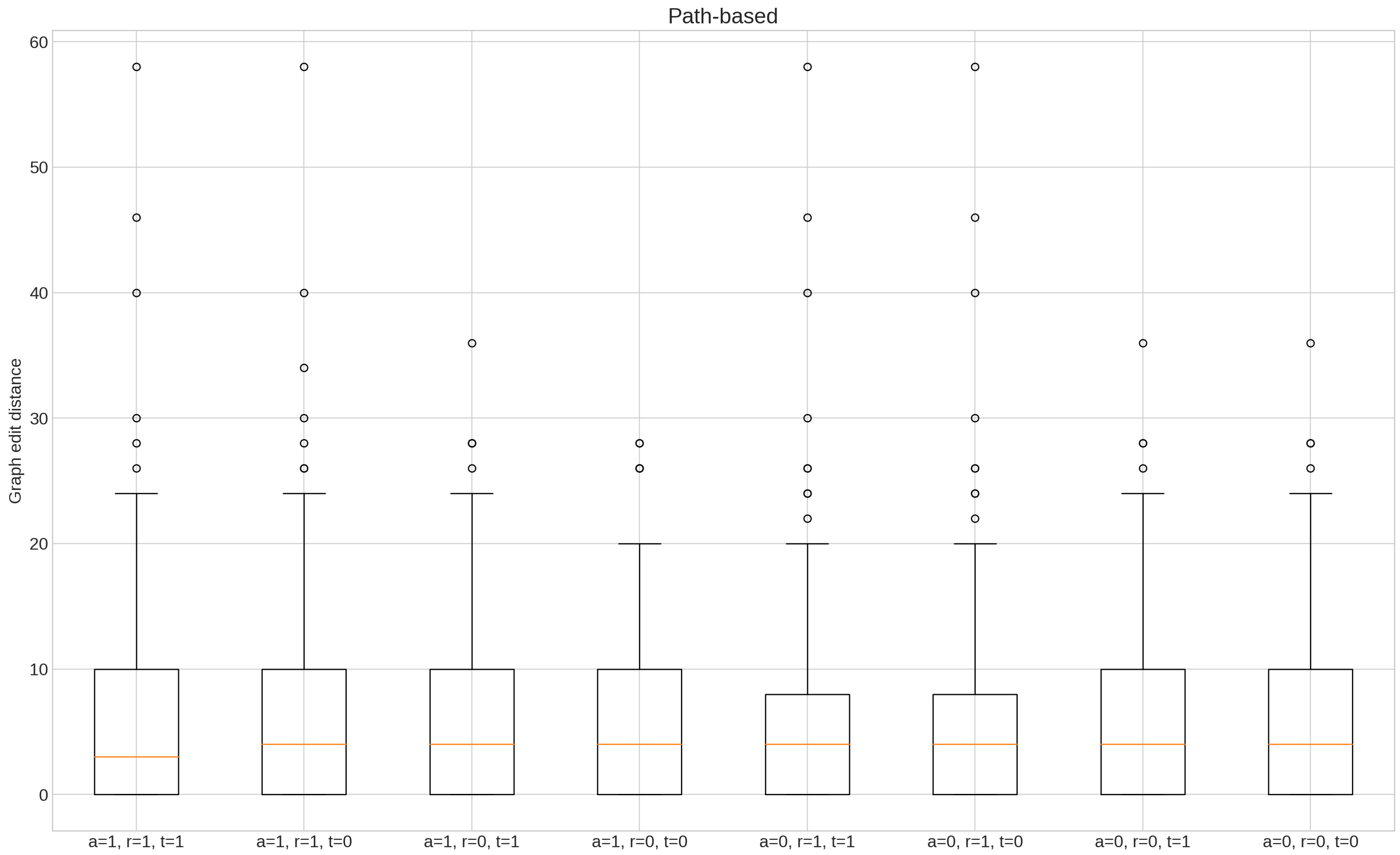

Figure 1: **Performance comparison of different ClonalTree parameter settings measured by GED path-based distances.** ClonalTree has three Boolean parameters, 'a' considers genotype abundances, 'r' adds unobserved internal nodes when necessary, and 't' tries to reduce the tree depth by performing attach/detach operations. "1" indicates that the parameter is true, while "0" is false.

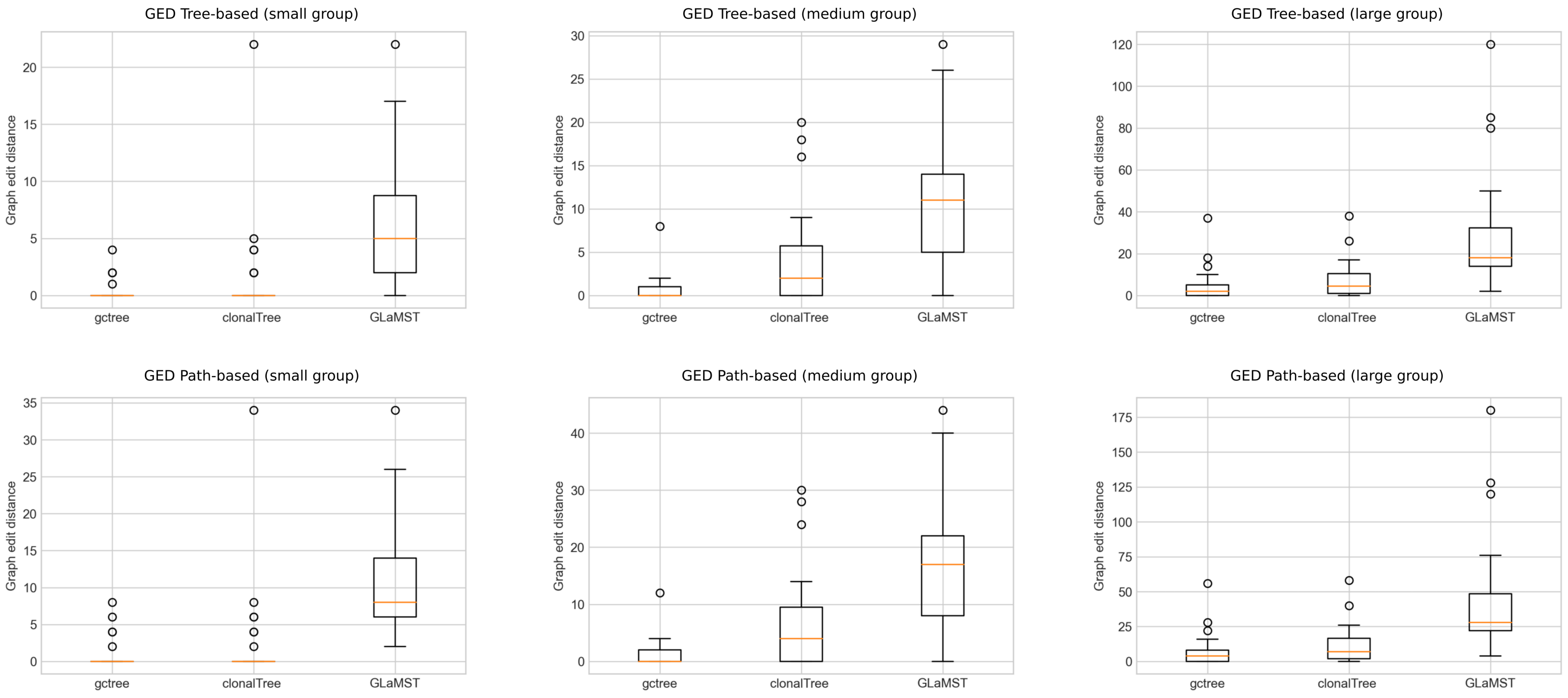

Figure 2: **Performance comparison among GCtree, ClonalTree, and GLaMST using GED distances on three categories of trees.** We split the trees into three categories according to their number of sequences: small (between 30 and 50), medium (between 60 and 80), and large (having more than 90 sequences).

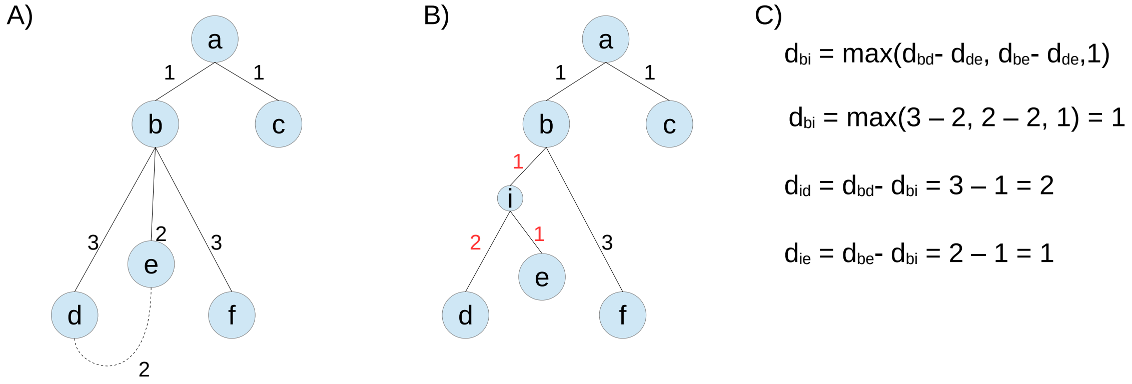

Figure 3: **Editing the reconstructed B cell lineage tree by adding unobserved internal nodes**. Unobserved internal nodes might represent unobserved sequences that were not sampled or disappeared during the affinity maturation When the distance between two sister nodes is smaller or equal to the distance to their parent (dotted line in A), we add an unobserved internal node as the common ancestor of the two sister nodes (node i in B). C shows how to update edge weights (in red).

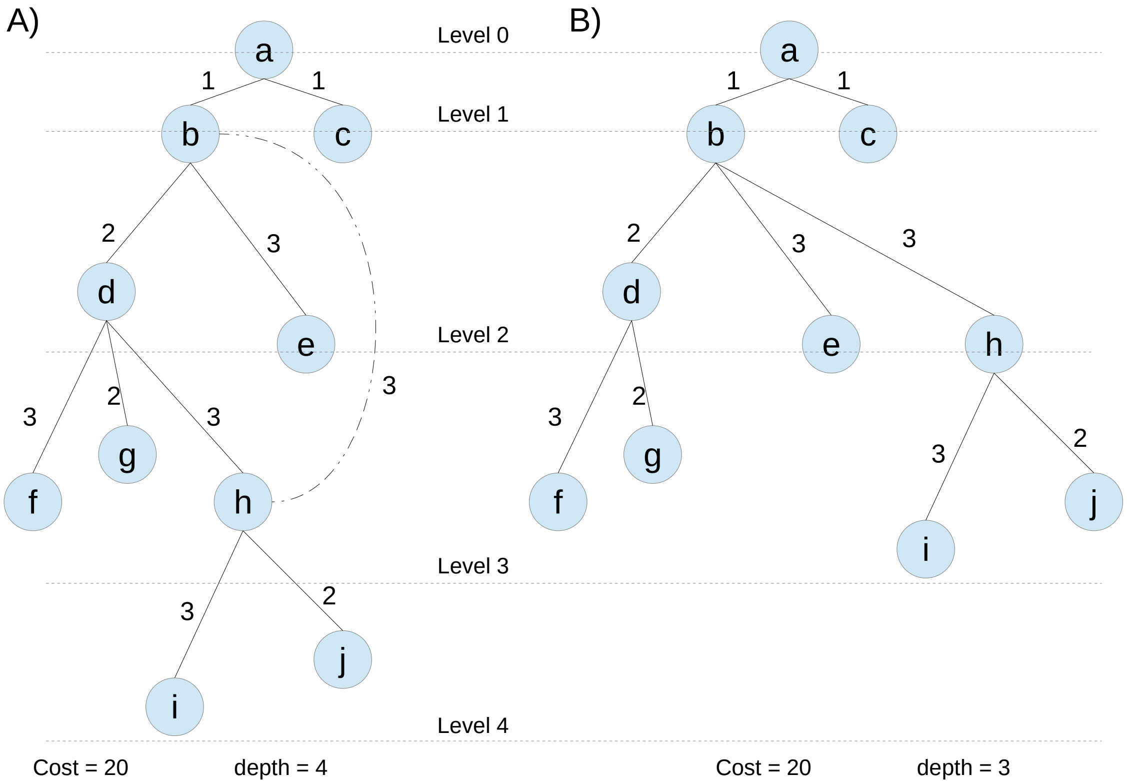

Figure 4: **Editing the reconstructed B cell lineage tree to reduce the depth of the tree while keeping its overall cost.** In this example, we transform tree (A) in tree (B) by performing a detach/attach operation. Note that the total cost of the tree, or the sum of edge weights, remained the same while the depth of it has been reduced. Edge weights represent the Hamming distance between sequences.

A)

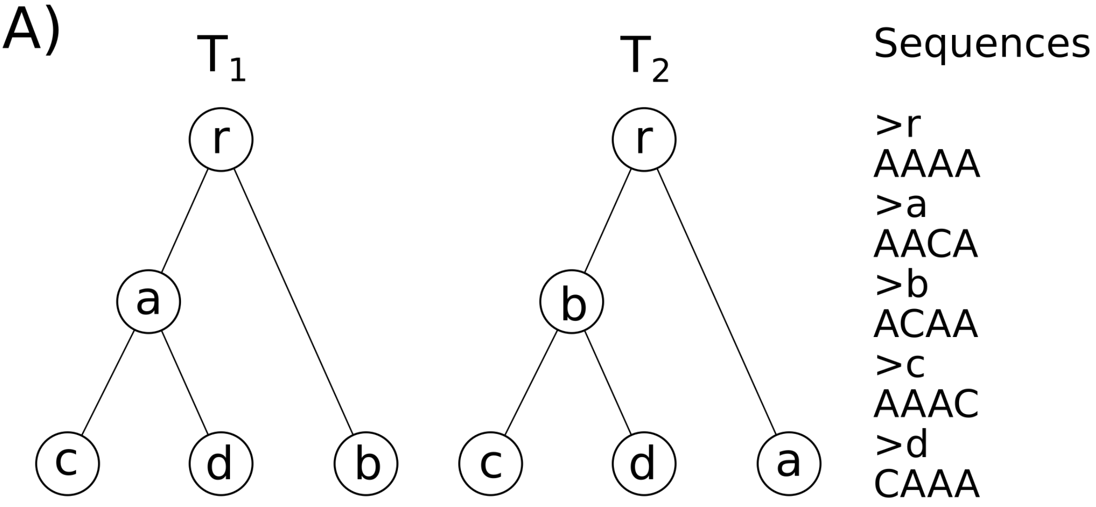

B)

Node pair(i,j)	Ancestral in T_1	Ancestral in T_2	MRCA(i,j)
c, d	a	b	2/4
c, a	a	r	1/4
c, r	r	r	0
c, b	r	b	1/4
d, a	a	r	1/4
d, b	r	b	1/4
d, r	r	r	0
a, r	r	r	0
a, b	r	r	0
b, r	r	r	0
		$\sum MRCA(i,j)$	6/4

C)

$$MRCA(T_1, T_2) = \frac{\sum MRCA(i,j)}{C_2^5} = \frac{6/4}{10} = 0.15$$

Figure 5: **An example of MRCA calculation.** A) Let's T_1 and T_2 be two comparable trees, and Sequences a fasta file containing nucleotide sequences associated to each node in both trees. B) For each pair of nodes $(i, j) \in T_1$ and $(i, j) \in T_2$, we find its most recent ancestral in both tree, and computed MRCA(i,j) as the normalized hamming distance bteween ancestral sequences. C) MRCA(T_1, T_2) is the average of MRCA(i,j) of all pair of nodes.

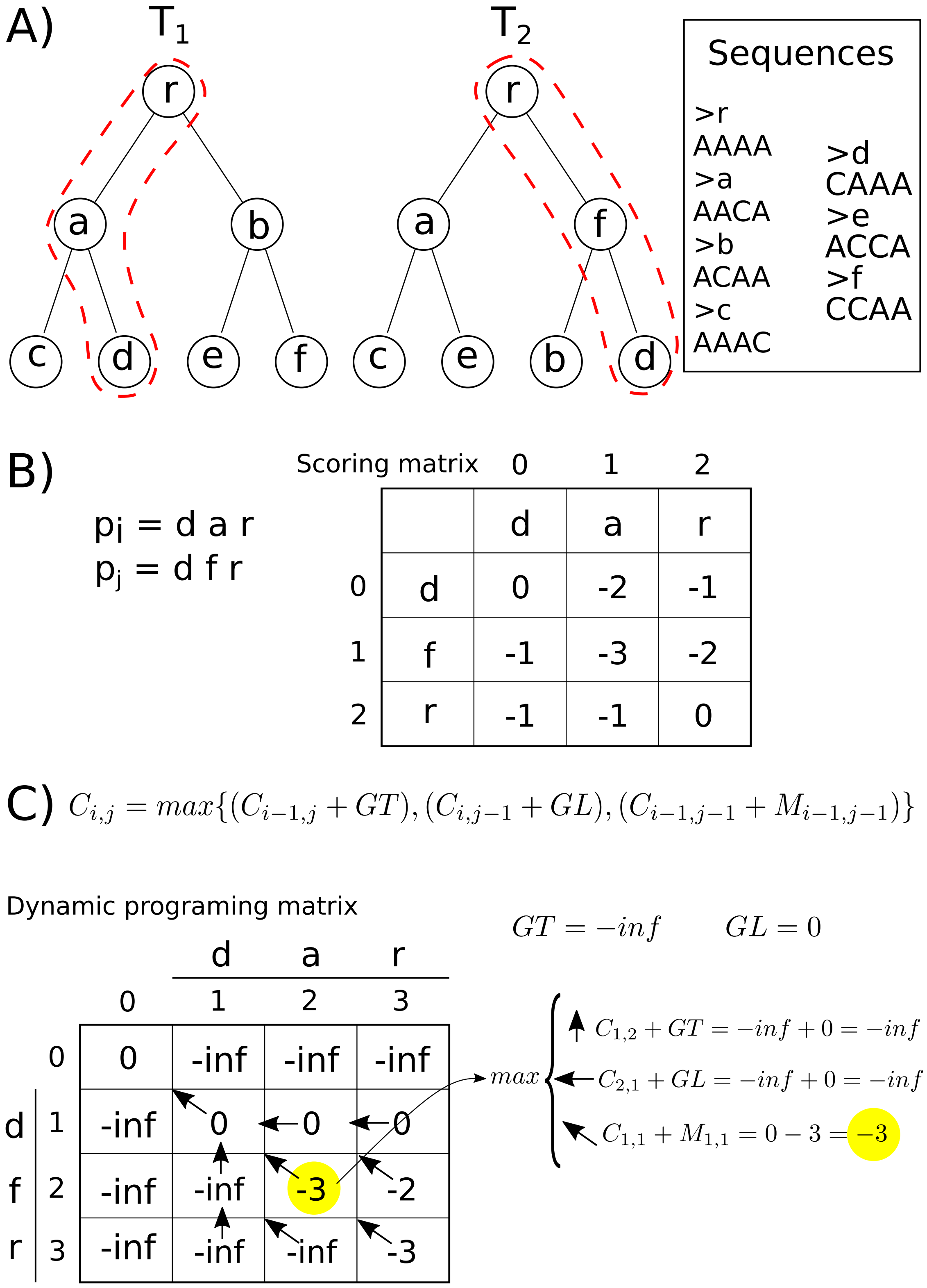

Figure 6: **An example of COAR calculation for a given leaf (see the algorithm below)** A) Let's T_1 and T_2 be two comparable trees, and Sequences a fasta file containing nucleotide sequences associated to each node in both trees. Let's dotted paths p_i and p_j represent comparable paths in T_1, and T_2, respectively. B) Paths to be compared and the scoring matrix containing negative hamming distances for each pair of sequence $S_i \in p_i$ and $S_j \in p_j$. C) We align paths with Needleman-Wunsch alignment algorithm, by using the scoring matrix computed in B, and special gap penalties: -inf for gap top (GT), and 0 for gap left (GL). Each element of the dynamic programming matrix is computed by the formula $C_{i,j}$, see an example for cell $C_{2,2}$. The COAR for the leaf i is 1=$\frac{minScore_i}{min(M)} = \frac{C_{3,3}}{-3} = \frac{-3}{-3}$.
