# Supplementary material for: Reconstructing B cell lineage trees with minimum spanning tree and genotype abundances
Source: BMC Bioinformatics. 2023 Feb 27;24:70. doi: 10.1186/s12859-022-05112-z (PMC9972711; doi:10.1186/s12859-022-05112-z)
Supplement: Supplementary file 2 — Additional file 2. COAR algorithm. [file 12859_2022_5112_MOESM2_ESM.pdf]

Reconstructing B cell lineage trees with minimum spanning tree and genotype abundances
Supplementary Algorithm

Nika Abdollahi[1,3], Lucile Jeusset[1,2], Anne de Septenville[2], Frédéric Davi[2] and Juliana Silva Bernardes[1,*]

[1]Sorbonne Université, CNRS, UMR 7238, Laboratoire de Biologie Computationnelle et Quantitative, Paris, France

[2]Sorbonne Université, AP-HP, Hôpital Pitié-Salpêtrière, Department of Biological Hematology, Paris, France

[3]IMGT®, the international ImMunoGeneTics Information System, CNRS, Institute of Human Genetics, Montpellier, France

[*]To whom correspondence should be addressed; E-mail: juliana.silva_bernardes@upmc.fr.

November 30, 2022

Algorithm 1: COAR algorithm

Require: T_1, T_2, sequences, GapPenalties
$COAR \leftarrow 0$
$N_L \leftarrow 0$ {Number of Leaves}
for all leaf $i \in T_1$ **do**
  $N_L \leftarrow N_L + 1$
  $p_i \leftarrow path(i, T_1)$
  $P \leftarrow paths(i, T_2)$ {take all paths in T_2 containing i}
  $minScore_i \leftarrow inf$
  {Compute a scoring matrix for sequences associated to nodes in $p_i \bigcup P$ based on negative hamming distances}
  $M \leftarrow scoreMatrix(P, p_i, sequences)$
  for all $p_j \in P$ **do**
    $scoreAln_i \leftarrow NWS(p_i, p_j, M, GapPenalties)$
    if $scoreAln_i > minScore_i$ **then**
      $minScore_i \leftarrow scoreAln_i$
    end if
  end for
  $COAR_i \leftarrow \frac{minScore_i}{min(M)}$
  $COAR \leftarrow COAR + COAR_i$
end for
return $\frac{COAR}{N_L}$

COAR algorithm requires two comparable trees T_1 and T_2, a set of nucleotide sequences, and Gap penalties. For each leaf $i \in T_1$, we find its path until the root, named p_i. We also compute P, a list of paths in T_2, containing i. Note that if i is a leaf in T_2, then $|P| = 1$, otherwise $|P| > 1$. We then compute a scoring matrix M for all nodes in $p_i \bigcup P$, each element of M contains the negative hamming distance between nodes' representative sequences. For instance, let a and b be nodes, and S_a='ACCA' and S_b='CCCC' the nucleotide sequences associated with those nodes. Then $M_{a,b}$ = -2. Next, for each $p_j \in P$, we align it with p_i to obtain the scoreAln$_i$. For that, we use the Needleman-Wunsch (NWS) algorithm, requiring the scoring matrix, previously computed, and Gap Penalties. In order to avoid gaps in the longest path, we use an NWS version that puts the longest sequence in the columns of the dynamic programming matrix and uses different gap penalties: 0 for the gap left (GL) and -inf for the gap top (GT). In case of $|P| > 1$, we keep the

minimum alignment score for i. Finally, we compute COAR_i as the ratio between minScore_i and the minimum value in M. We iterate until we compute COAR_i for all $i \in T_1$, and we return the average: the overall COAR divided by the number of leaves in T_1. We ilustrate a iteration of COAR algorithm above.
